# Triglyceride and Small Dense LDL-Cholesterol in Patients with Acute Coronary Syndrome

**DOI:** 10.3390/jcm10194607

**Published:** 2021-10-08

**Authors:** Masakazu Hori, Teruhiko Imamura, Nikhil Narang, Hiroshi Onoda, Shuhei Tanaka, Ryuichi Ushijima, Mitsuo Sobajima, Nobuyuki Fukuda, Hiroshi Ueno, Koichiro Kinugawa

**Affiliations:** 1 The Second Department of Internal Medicine, University of Toyama, Toyama 9300072, Japan; masahori6059@yahoo.co.jp (M.H.); ohiro0203@gmail.com (H.O.); stanaka@med.u-toyama.ac.jp (S.T.); ryuryu0702@gmail.com (R.U.); soba1126@yahoo.co.jp (M.S.); nfukuda@med.u-toyama.ac.jp (N.F.); hueno@med.u-toyama.ac.jp (H.U.); kinugawa-tky@umin.ac.jp (K.K.); 2 Advocate Christ Medical Center, Oak Lawn, IL 60453, USA; nikhil.narang@gmail.com

**Keywords:** dyslipidemia, coronary artery disease, obesity

## Abstract

Background: Small dense LDL-cholesterol is an established risk factor for atherosclerosis but is not routinely measured in daily practice. The association between small dense LDL-cholesterol and triglyceride, which in turn is routinely measured, in patients with acute coronary syndrome remains unknown. Methods: Consecutive patients with acute coronary syndrome who were admitted to our institute were prospectively included, and serum samples were obtained on admission. The association between small dense LDL-cholesterol and triglyceride was investigated. Results: Among 55 patients (median 71 years old, 64% men), median (interquartile range) small dense LDL-cholesterol was 23.6 (17.0, 36.0) and triglyceride was 101 (60, 134) mg/dL. Triglyceride level correlated with small dense LDL-cholesterol (*r* = 0.67, *p* < 0.001) and was an independent determinant of small dense LDL-cholesterol together with body mass index (*p* = 0.010 and *p* = 0.008, respectively). Those with high triglyceride and high body mass index had a 3-fold level of small dense LDL-cholesterol compared with those with low triglyceride and low body mass index (45.8 [35.0, 54.0] mg/dL versus 15.0 [11.6, 23.7] mg/dL, *p* = 0.001). Conclusions: Triglyceride level was a major determinant of small dense LDL-cholesterol in patients with acute coronary syndrome. Triglyceride level might be a useful and practical biomarker for risk stratification for patients with acute coronary syndrome together with body mass index.

## 1. Background

As one of the lipid parameters, small dense LDL-cholesterol is being measured more often while being incorporated into clinical risk assessment tools as a research basis [1]. Small dense LDL-cholesterol has a large specific gravity and is a small particle among the LDL-cholesterol subtypes. Small dense LDL-cholesterol is a risk factor of cardiovascular diseases independent of LDL-cholesterol levels [2]. A higher small dense LDL-cholesterol level is associated with incremental risk of cardiovascular diseases even among those with LDL-cholesterol < 100 mg/dL [3].

Small dense LDL-cholesterol is also strongly associated with accelerated progression of atherosclerosis [4], due to stronger endothelial cell adhesion owing to its small size and its vulnerability to oxidization.

Triglyceride levels are commonly measured in clinical lipid profiles. Triglyceride levels are not typically associated with atherosclerosis progression, but are known to be associated with the presence of small dense LDL-cholesterol [5]. The number of small dense LDL-cholesterol particles, in theory, may increase in concordance with triglyceride levels, particularly when the level of LDL-cholesterol is high [6]. Knowing this, high triglyceride levels through the interaction with a higher burden of small dense LDL-cholesterol were shown to increase the risk of coronary disease, comparable to those of LDL-cholesterol, in the JDCS study [7].

Given a prognostic implication of small dense LDL-cholesterol and prior observed associations between small dense LDL-cholesterol level and triglyceride level, triglyceride levels not only provide incremental data on clinical risk but also are a target for cardiovascular disease risk reduction [8]. Of note, small dense LDL-cholesterol cannot be measured routinely in most clinical laboratories as it is non-reimbursable. The association between small dense LDL-cholesterol and triglyceride has been reported in low-risk cohorts [9]. However, their association among those with acute coronary syndrome, which would be more important to consider for additional targets in secondary prevention, has not yet been investigated. In this study, we investigated the association between small dense LDL-cholesterol and triglyceride in patients with acute coronary syndrome.

## 2. Methods

### 2.1. Patient Selection

Consecutive patients who were admitted to our institute with a diagnosis of acute coronary syndrome between Jun 2016 and May 2021 were prospectively included. Acute coronary syndrome was diagnosed considering symptoms, echocardiography, electrocardiogram, and laboratory data [10]. Data acquisition and analysis was performed in compliance with protocols approved by the Ethical Committee of the University of Toyama (ethical approval number R2015154). Written informed consent was obtained from all participants beforehand.

### 2.2. Biomarker Measurement

Lipid parameters including triglyceride were assayed by institutional standard laboratory procedures using automated analyzer kits (MetaboLead or Determiner (Minaris Medical Co. Ltd., Tokyo, Japan)). All serum samples were obtained just on admission before heparin infusion at fasting condition and frozen at −80 degrees Celsius immediately. Small dense LDL-cholesterol level was measured at the external laboratory institute (BML, Inc., Tokyo, Japan).

### 2.3. Other Clinical Data Obtained

Demographics, comorbidities, and other standard laboratory and medication data obtained on admission were retrieved as baseline characteristics.

### 2.4. Statistical Analysis

Continuous variables were expressed as median and interquartile range irrespective of their distribution. Categorical variables were expressed as numbers and percentages. Correlation between small dense LDL-cholesterol and other lipid data were assessed by Pearson’s correlation coefficient. Of note, the primary outcome of interest was a correlation between small dense LDL-cholesterol and triglyceride. Linear regression analyses were performed to investigate clinical variables associated with small dense LDL-cholesterol levels. Variables with *p* < 0.05 in the univariate analyses were included in the multivariate analysis. A 2-sided value of *p* < 0.05 was considered statistically significant. Statistical analyses were performed using SPSS Statistics 22 (SPSS Inc, Armonk, IL, USA).

## 3. Results

### 3.1. Baseline Characteristics

**Figure 1:**
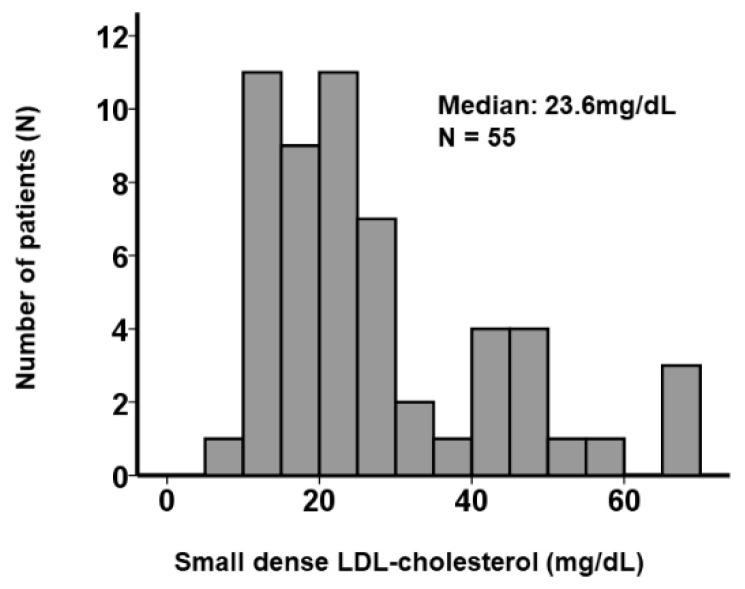
Distribution of small dense LDL-cholesterol.

A total of 55 ACS patients who were admitted to our institute were included (Table 1). Median age was 71 (62, 84) years old and 64% were men. Forty percent had a history of diabetes mellitus. Median small dense LDL-cholesterol was 23.6 (17.0, 36.0) mg/dL. Small dense LDL-cholesterol was widely distributed (Figure 1). Median triglyceride was 101 (60, 134) mg/dL and median LDL-cholesterol was 116 (76, 131) mg/dL. Thirty-three percent had a history of statin use on admission.

### 3.2. Association between Small Dense LDL-Cholesterol and Other Lipid Markers

**Figure 2:**
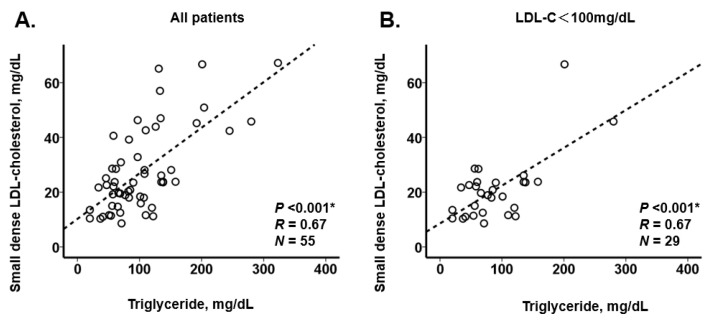
Association between triglyceride and small dense LDL-cholesterol in the overall cohort (*N* = 55); (**A**) and those with LDL-cholesterol < 100 mg/dL (*N* = 29); (**B**). * *p* < 0.05 by Pearson’s correlation coefficient.

**Figure 3:**
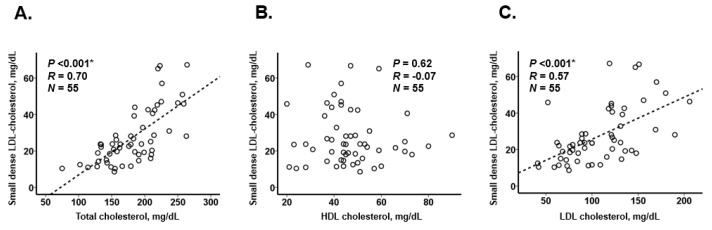
Association between small dense LDL-cholesterol and total cholesterol (**A**), HDL-cholesterol (**B**), and LDL-cholesterol (**C**). * *p* < 0.05 by Pearson’s correlation coefficient.

We observed a positive correlation between small dense LDL-cholesterol and triglyceride level (*R* = 0.67; Figure 2A). A positive correlation remained even among those with LDL-cholesterol < 100 mg/dL (*R* = 0.67; Figure 2B). If we used a cutoff of small dense LDL-cholesterol 20.9 mg/dL [11], a corresponding cutoff of triglyceride was calculated as 62.9 mg/dL. A moderately positive correlation was observed between small dense LDL-cholesterol and other lipid parameters, except for HDL-cholesterol (Figure 3A–C).

In a sub-group with statin therapy (*N* = 18), there was a significant correlation between triglyceride and small dense LDL-cholesterol (*r* = 0.544, *p* = 0.020). There was a similar trend among those without statin therapy (*N* = 37, r = 0.693, *p* < 0.001). There was a significant correlation between triglyceride and small dense LDL-cholesterol in patients with diabetes mellitus (*r* = 0.708, *p* < 0.001, N = 22) and without diabetes mellitus (*r* = 0.705, *p* < 0.001, *N* = 33).

### 3.3. Association between Small Dense LDL-Cholesterol and Clinical Variables

Several clinical parameters including age were associated with small dense LDL-cholesterol (*p* < 0.05; Table 2). Following the adjustment for variables significant in the univariate analyses, body mass index and triglyceride were independent statistical determinants of small dense LDL-cholesterol (*p* = 0.008 and *p* = 0.010, respectively).

### 3.4. Small Dense LDL-Cholesterol, Triglyceride, and Body Mass Index

**Figure 4:**
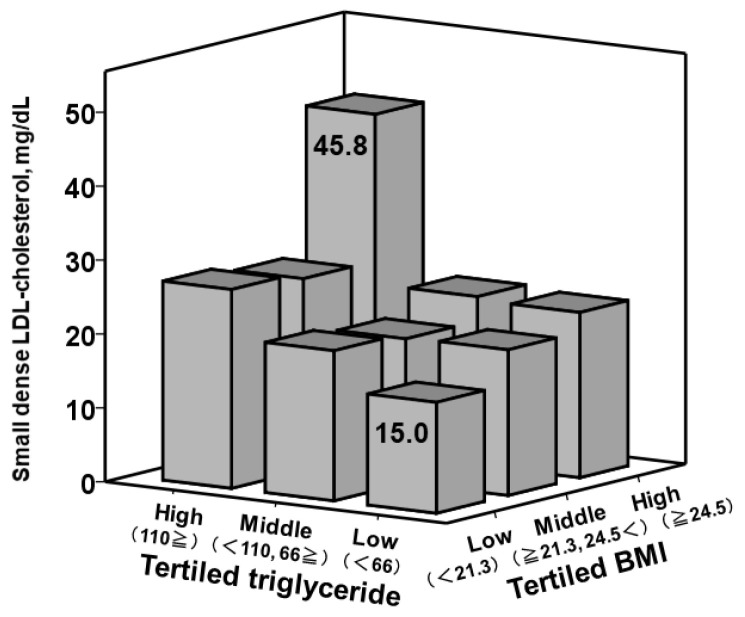
Median small dense LDL-cholesterol levels stratified by the tertiled triglyceride and BMI levels. BMI, body mass index.

Small dense LDL-cholesterol levels were stratified into the nine groups by the tertiled triglyceride and tertiled body mass index (Figure 4). Even though the triglyceride level was low (i.e., the first tertile), small dense LDL-cholesterol level was higher in those with higher body mass index (*N* = 19, *p* = 0.18). The highest level of small dense LDL-cholesterol was observed in patients with high triglyceride and high body mass index, with a 3-fold level as compared with the low triglyceride and low body mass index group (45.8 [35.0, 54.0] mg/dL versus 15.0 [11.6, 23.7] mg/dL; *p* = 0.001).

## 4. Discussion

In this study, we investigated the association between small dense LDL-cholesterol and triglyceride in patients with acute coronary syndrome. The major findings are as follows: (1) Small dense LDL-cholesterol and triglyceride positively correlated among those with acute coronary syndrome; (2) Triglyceride level was an independent determinant of small dense LDL-cholesterol level together with body mass index.

### 4.1. Small Dense LDL-Cholesterol and Triglyceride in Acute Coronary Syndrome

In our study, although conducted among a small sample-sized cohort, triglyceride level had a positive correlation with small dense LDL-cholesterol among those with acute coronary syndrome. Given that small-dense LDL-cholesterol is a risk factor for the development and progression of atherosclerosis [11] and that this important parameter cannot be routinely measured given challenges with insurance reimbursement in Japan, we might be able to utilize triglyceride as a promising marker in secondary risk prevention.

Of note, we observed such a positive correlation among those with acute coronary syndrome. Previous studies have reported their association among those with a history of diabetes mellitus and coronary artery disease [9]. However, these prior reports cannot be generalized in those with acute coronary syndrome.

There is no established cutoff of small dense LDL-cholesterol for risk stratification. Several studies found that small dense LDL-cholesterol was elevated in patients with acute coronary syndrome [12,13]. Recently, Sekimoto and colleagues demonstrated that a small dense LDL-cholesterol > 20.9 mg/dL was associated with higher incidence of adverse events following acute coronary syndrome [11]. Another recent study also demonstrated that the elevated small dense LDL-cholesterol was a risk factor of future cardiovascular events following acute coronary syndrome [14]. Given our findings (Figure 2A), a cutoff of triglyceride 200 mg/dL, which is a cutoff currently recommended by the guidelines [10], might not be sufficient as a marker for accurate risk prediction in secondary prevention. Even though LDL-cholesterol is well controlled under < 100 mg/dL, triglyceride level remains as a potentially useful surrogate of small dense LDL-cholesterol (Figure 2B).

### 4.2. Triglyceride and Body Mass Index as Determinants of Small Dense LDL-Cholesterol

Triglyceride levels were independently associated with the small dense LDL-cholesterol burden along with body mass index. Hirano and colleagues also reported that higher levels of triglyceride were associated with smaller-sized LDL particles [5], i.e., small dense LDL-cholesterol. Insulin resistance, which is often seen in patients with metabolic syndrome where there is a high prevalence of obesity, is also associated with a higher burden of small dense LDL-cholesterol [9].

We believe that triglyceride levels can act as a surrogate marker of small dense LDL-cholesterol to risk-stratify the patients with acute coronary syndrome. Given our findings, a relatively low triglyceride level (i.e., between 100 and 150 mg/dL) might not represent true lower freedom from cardiovascular events given a potential unmeasured interaction with small dense LDL-cholesterol. Consistently, we calculated a cutoff of triglyceride 62.9 mg/dL, which was statistically equivalent to 20.9 mg/dL of small dense LDL-cholesterol—a proposed cutoff to risk-stratify those with acute coronary syndrome [11]. Furthermore, closer attention must be given to obese patients given the additional risk of elevated small dense LDL-cholesterol independent of triglyceride levels. Given all together, those with relatively lower triglyceride (between 100 and 150 mg/dL) and/or obesity might be good candidates for further aggressive efforts to decrease triglyceride or assay of small dense LDL-cholesterol if available.

### 4.3. Limitations

This study consists of a small sample cohort. We should be careful not to interpret non-significant findings in this study. We lacked a control group and just investigated the association between small dense LDL-cholesterol and triglyceride as well as other lipid parameters. The prognostic impact of these variables and the implication of any interventions to these variables needs further investigation.

## 5. Conclusions

Triglyceride levels significantly correlated with small dense LDL-cholesterol in patients with acute coronary syndrome. Triglyceride levels may be a useful biomarker for risk stratification in patients with acute coronary syndrome together with body mass index, where small dense LDL-cholesterol level burden is higher.

## Figures and Tables

**Table 1:** Baseline characteristics.

	*N* = 55
Demographics	
Age, years	71 (62, 84)
Male	35 (64%)
Body mass index	23.6 (21.9, 26.4)
ST elevation MI/Non-ST elevation MI	37/18
Comorbidity	
Diabetes mellitus	22 (40%)
Chronic kidney disease	20 (36%)
History of stroke	8 (15%)
History of myocardial infarction	4 (7%)
History of heart failure admission	4 (7%)
Current smoking	35 (66%)
Atrial fibrillation	6 (11%)
Echocardiography	
Left ventricular end-diastolic diameter, mm	48 (44, 49)
Left ventricular ejection fraction, %	53 (45, 60)
Laboratory	
Hemoglobin, g/dL	13.5 (12.0, 14.5)
Serum albumin, g/dL	3.8 (3.4, 4.2)
Serum sodium, mEq/L	139 (137, 140)
Serum uric acid, mg/dL	6.3 (4.8, 8.0)
Blood glucose g/dL	148 (119, 207)
NTpro B-type natriuretic peptide, pg/mL	828 (182, 2700)
eGFR, mL/min/1.73 m^2^	61.1 (36.7, 73.9)
Hemoglobin A1c, %	5.8 (5.7, 6.6)
Triglyceride, mg/dL	101 (60, 134)
Total cholesterol, mg/dL	184 (151, 214)
HDL cholesterol, mg/dL	45 (41, 49)
LDL cholesterol, mg/dL	116 (76, 131)
Small dense LDL-cholesterol, mg/dL	23.6 (17.0, 36.0)
Medication	
Beta-blocker	10 (18%)
Renin angiotensin system inhibitor	24 (44%)
Mineralocorticoid receptor blocker	4 (7%)
Statin	18 (33%)

MI, myocardial infarction; eGFR, estimated glomerular filtration ratio. Continuous variables were expressed as median and interquartile. Categorical variables were expressed as number and percentage.

**Table 2:** Association between small dense LDL-cholesterol and other clinical parameters including triglyceride.

	Univariate Analysis		Multivariate Analysis	
	Beta Value	*p* Value	Beta Value	*p* Value
Age, years	−0.45	0.004 *	−0.05	0.69
Male	4.93	0.25		
Body mass index	1.78	<0.001 *	1.06	0.008 *
Diabetes mellitus	−1.08	0.80		
Chronic kidney disease	−2.31	0.59		
Current smoking	5.02	0.26		
Atrial fibrillation	−0.19	0.98		
Left ventricular end-diastolic diameter, mm	0.46	0.22		
Left ventricular ejection fraction, %	0.35	0.006 *	0.05	0.63
Hemoglobin, g/dL	2.40	0.006 *	−0.12	0.89
Serum albumin, g/dL	9.23	0.013 *		
Serum sodium, mEq/L	0.007	0.99		
Serum uric acid, mg/dL	0.029	0.97		
Blood glucose g/dL	−0.016	0.31		
NTpro B-type natriuretic peptide, pg/mL	0.001	0.22		
eGFR, mL/min/1.73 m^2^	0.17	0.040 *		
Triglyceride, mg/dL	0.17	<0.001 *	0.08	0.010 *
Total cholesterol, mg/dL	0.25	<0.001 *	0.14	0.058
HDL cholesterol, mg/dL	−0.073	0.62		
LDL cholesterol, mg/dL	0.23	<0.001 *	0.05	0.49
Statin use	−6.50	0.14		

eGFR, estimated glomerular filtration ratio. * *p* < 0.05 by linear regression analysis. Variables with *p* < 0.010 in the univariate analyses were included in the multivariate analysis.

## Data Availability

Data are available from the corresponding authors upon reasonable requests.
